# Assessing Resident Palliative Care Education: Lessons Learned for Curriculum Mapping in Graduate Medical Education

**DOI:** 10.15694/mep.2018.0000259.1

**Published:** 2018-11-14

**Authors:** Ashwini Bapat, Matthew Ellman, Laura Morrison

**Affiliations:** 1 Division of Palliative and Geriatric Medicine; 2 Department of Internal Medicine; 3 Yale Palliative Care Program

**Keywords:** Graduate Medical Education, Curriculum Mapping, Palliative Care, End-of-Life Care

## Abstract

This article was migrated. The article was marked as recommended.

Charged with implementing a new curriculum within an established residency, we describe the application of curriculum mapping, a tool underutilized in GME. As proof of concept, we utilized curriculum mapping to identify existing palliative care didactic content and deficiencies within the Yale Internal Medicine Residency Programs for one academic year. Mapping included three steps: determining core educational venues, identifying and analyzing didactic content, and aligning content with published competencies. The curriculum map contained data for 5 of 9 educational venues, demonstrating gaps in Terminal Care & Bereavement, Spirituality, and Hospice Care. These gaps have informed the new palliative care curriculum. Although curriculum mapping has potential for application in GME, it is limited by available data.

## Introduction

We were charged with designing a comprehensive palliative care curriculum for internal medicine (IM) residents at our institution. The appeal for primary palliative care competency in all health care providers has focused on implementation of formal curricula ( Schaefer *et al.*, 2014), though the literature documents few residency level initiatives ( Schaefer *et al.*, 2014; Medicine, 2015). Without a required palliative care rotation, it is difficult to determine if a trainee’s exposure to palliative care is satisfactory. Although needs assessment surveys of learners aid in defining gaps and identifying areas of strength, it is unclear if deficiencies exist because a topic is not covered or taught ineffectively. We identified curriculum mapping as a data-driven approach to inventory our existing palliative care exposures.

A curriculum is a blend of educational strategies, content, learning outcomes, and assessments. Curriculum mapping makes the links between these components transparent to learners, educators, and administrators ( Harden, 2001). Mandated by Accreditation Council for Graduate Medical Education each residency submits an educational curriculum ( Education, 2016). Operational delivery of a specific curriculum remains a challenge ( Wong and Roberts, 2007). Through curriculum mapping, a training program could align the declared, taught, and experienced curriculum, preventing gaps and unintentional repetition ( Harden, 2001). The data utilized in a curriculum map includes syllabus review, faculty and student self-reports, and assessments ( Plaza *et al.*, 2007). American medical schools use curriculum mapping to meet Liaison Committee on Medical Education accreditation; it is also utilized by the majority of Canadian and UK medical schools ( Willett, 2008). Promoted as an essential tool for curricular development and implementation ( Harden, 2001) and shown to improve medical school ( Meekin *et al.*, 2000; Wachtler and Troein, 2003) and residency education ( Harden, 2001; Wong and Roberts, 2007) curriculum mapping appears underutilized in GME.

We applied curriculum mapping within GME to define the state of palliative care didactic education in the Yale IM Residency Programs: Primary Care (YPC) and Traditional (IM-Trad). We describe the development of our curriculum map, the challenges encountered, and lessons learned.

## Methods

For July 1, 2014 to June 30, 2015, we identified and aligned the palliative care didactic content within YPC and IM-Trad with five published residency competency domains: Pain & Symptom Management, Communication, Psychosocial/Spiritual/Cultural Aspects of Care, Terminal Care & Bereavement (TCB), Palliative Care Principles & Practice. ( Schaefer *et al.*, 2014) We focused on didactic content since it is a good measure of instructional content ( Porter, 2002), is mandated by the ACGME ( Education, 2016), and remains the most common way to disseminate medical knowledge ( Sawatsky *et al.*, 2014). In line with many curriculum mapping methods, didactic content served as a proxy for educational content acquired in direct patient care ( Arafeh, 2016).

We mapped the palliative care didactic content in 3 steps. First, we determined core didactic venues: morning report, attending rounds, noon conference, IM grand rounds, academic half days, geriatrics required rotation, electives, Yale IM intranet curriculum, intern orientation, outpatient pre-clinic conference based on Yale Office-Based Medicine (YOBM) 8 ^th^ Edition (Rosenbaum JR *et al.,*). Each entry in our map met the following criteria: available title and/or learning objectives, identification of covered palliative care competency, and potential for interaction between educator and learner. The first two criteria reflect the minimal information needed for the map to be meaningful and the third emphasizes opportunities for active participation, improving resident learning ( Sawatsky *et al.*, 2014).

Second, we identified and analyzed the didactic content within each venue. We determined the content of morning report, attending rounds, and noon conference by reviewing the resident conference calendar and by emailing chief residents and faculty of both programs. We requested the title and/or learning objectives of each didactic, provided a list of the five palliative care competency domains, and asked the faculty to identify which if any domain was covered. Non-responders received a reminder email. We reviewed the content of IM grand rounds available on the Yale School of Medicine website, academic half days available on Yale MedHub ^TM^ and in administrative records, Geriatrics required rotation and electives available on Yale MedHub ^TM^, Yale IM Intranet curriculum, and intern orientation in administrative records. The outpatient pre-clinic conference based on YOBM was previously reviewed ( Bournival N *et al.*, 2015) and was not included in our map. Third, we mapped content to the competency domains, creating a curriculum map ( Table 1).

**Table 1.** Curriculum Map of Didactic Exposure to Palliative Care

Educational Venues	Pain & Symptom Management	Communication	Psychosocial/Spiritual/Cultural Aspects	Terminal Care & Bereavement	Palliative Care Principles &Practices
**GRAND ROUNDS**					
Communication: The Patient & Provider Experience		**X**			
Many Sides of a Veterans Cavitary Lesion		**X**			
**MORNING REPORT**					
Acute Intermittent Porphyria	**X**	**X**			
Angioedema Due To Medications		**X**			
Hypoglycemia and Pain Due To Metastatic Cancer	**X**	**X**	**X**		**X**
Dementia		**X**	**X**		
Acute HIV		**X**			
Resistant Secondary Hypertension			**X**		
Tumor Lysis Syndrome					**X**
**ATTENDING ROUNDS**					
Postural Orthostatic Tachycardia Syndrome		**X**	**X**		
Bleeding Peptic Ulcer	**X**				
Acute Pancreatitis	**X**	**X**	**X**		
UTI in Elderly	**X**		**X**		
Role of Procalcitonin in Pneumonia		**X**			
Dysphagia Evaluation	**X**		**X**		
Cannabinoid Hyperemesis	**X**	**X**	**X**		
Medicare and Medicaid			**X**		**X**
Hemochromatosis		**X**			
Dysphagia in the Elderly	**X**	**X**	**X**		**X**
VTE Treatment	**X**				
Observation Status			**X**		
ACA HealthCare Exchanges		**X**	**X**		
Prolonged Hospitalization &Poor Wound Healing	**X**				
Caregiver Burnout		**X**	**X**		
Bedside Presentations		**X**	**X**		
Goals of Care	**X**	**X**	**X**		**X**
Chronic Pain	**X**				
PE and Patient Preference		**X**	**X**		
Home Visits		**X**	**X**		
Type II Diabetes		**X**	**X**		
Leukemia in Elderly		**X**			**X**
PE Diagnosis		**X**	**X**		
Pain Management	**X**				
Family Presence During CPR		**X**	**X**		
Decision Making Capacity		**X**			
**ACADEMIC HALF DAYS**					
Patient Centered Interviewing			**X**		
Ethics/Policy Day			**X**		
Transitions Day		**X**			
Communication Curriculum: Introduction to Smith’s 5 Step Patient Centered Interview		**X**			
**INTERN ORIENTATION**					
Goals of Care		**X**	**X**		

## Results/Analysis

Within YPC, with 5 chief residents and 17 faculty, we achieved 13 email responses, 10 providing data meeting mapping criteria. Within IM-Trad, we emailed the 5 chief residents, discovering that no consistent record of teaching for morning report, attending rounds, and noon conference existed, preventing identification of didactics. Emailing faculty within IM-Trad was infeasible given the number involved and difficulty identifying which faculty taught specific didactics. The resident conference calendar revealed incomplete information with missing titles and/or learning objectives. Within both programs, the content of noon conferences (available in YPC only), Geriatrics rotation, electives, and Yale Intranet Curriculum did not meet inclusion criteria. Titles and learning objectives were absent for noon conferences and Geriatrics rotation. The Yale Intranet Curriculum listed a set of palliative care readings without opportunity for educator and learner interaction.

Our curriculum map contained data for 5 of the 9 educational venues ( Table 1), identifying 40 didactics addressing at least one palliative care competency domain. Significant deficits surfaced for TCB, Spirituality, and Hospice Care. Results from two venues, morning report and attending rounds were exclusively within YPC, accounting for 33 didactics.

## Discussion

We applied curriculum mapping in our GME setting, as an illustrative example, identifying both challenges in the process and a trend toward important educational gaps. The discussion below will focus in on the curriculum mapping process.

### Challenges

The lack of a central educational database, ubiquitous amongst residency programs ( Wong and Roberts 2007), limited the comprehensiveness of our curriculum map. Even when email inquiry provided data, response accuracy was likely influenced by respondent recall and interpretation of the competency domains. Though the contribution of non-didactic education was beyond the scope of this study, this data should also be explored. We obtained more complete data from YPC which promotes a biopsychosocial model of patient care, perhaps not accurately reflecting the curriculum in the IM-Trad program, and over representing the palliative care content. Data interpretation was challenging. As is often the case with competency-based curriculum mapping of didactics ( Arafeh, 2016) we were unable to assess if key knowledge, skills, and attitudes were meaningfully conveyed.

### Lessons Learned

Ideally, curriculum mapping would be used in real-time within a competency-based framework to monitor, assess, and modify curricula, verifying that trainees achieve developmental milestones ( Harden, 2001; Wong and Roberts, 2007). A robust curriculum assessment should include learner outcomes (our IM resident palliative care needs assessment survey will be published elsewhere) but would require more dependable data. Creating and maintaining a central didactic repository would require administrative and faculty assistance, though residencies could provide more detailed content in their electronic teaching calendars.

## Conclusion

Through curriculum mapping, we identified didactic content gaps, informing a new resident palliative care curriculum.

## Take Home Messages

• Curriculum Mapping is underutilized in Graduate Medical Education
• Curriculum Mapping requires a competency based framework
• Curriculum Mapping helped uncover important educational gaps
• The lack of a central educational database, ubiquitous amongst residency programs, limited the comprehensiveness of our curriculum map

## Notes On Contributors

Dr. Bapat is an Instructor in Medicine at Harvard Medical School and a Palliative Care Physician at Massachusetts General Hospital, Boston, MA.

Dr. Ellman is Associate Professor of Medicine and Director of Medical Student Palliative and End-of-Life Care Education, Yale University School of Medicine, New Haven, CT.

Dr. Morrison is Associate Professor of Medicine, Director of Hospice and Palliative Medicine Education, Director of Hospice and Palliative Medicine Fellowship, New Haven, CT.
