# Supplementary material for: Assessing Resident Palliative Care Education: Lessons Learned for Curriculum Mapping in Graduate Medical Education
Source: MedEdPublish (2016). 2018 Nov 14;7:259. [Version 1] doi: 10.15694/mep.2018.0000259.1 (PMC10711968; doi:10.15694/mep.2018.0000259.1)
Supplement: Peer review history

# Reviewer report: Reviewer response for version 1

**DOI:** 10.21956/mep.19713.r29500

**Published:** 2019-03-26

**Authors:** Trevor Gibbs

**Affiliations:** 1 AMEE

This review has been migrated. The reviewer awarded 3 stars out of 5 Thank you for asking me to review this paper. The development of a curriculum map is not a new concept and I do not feel that this paper added much to how we develop a curriculum map. I too found it a rather difficult paper to read and was lost sometimes in the over use of abbreviations that were not explained properly. My observation from this paper was that an effective use of a curriculum map can highlight gaps in the teaching, learning and eventually the assessment process.

Reviewer Expertise:

NA

No decision status is available

# Reviewer report: Reviewer response for version 1

**DOI:** 10.21956/mep.19713.r29499

**Published:** 2019-03-25

**Authors:** Richard Hays

**Affiliations:** 1 James Cook University

This review has been migrated. The reviewer awarded 4 stars out of 5 This is not an easy paper to review, perhaps because it is about technical 'backroom' educational development that does not attract much attention. There is also some terminology that is unique to the North American context, so the authors might like to consider using more generic terms, or explaining some terms , to improve meaning in other countries. However, I would like to commend the authors for demonstrating a strong principle of curriculum design. That is, there needs to be a road map that connects learners and teachers alike to curriculum content and how it relates to the surrounding environment. There are often several somewhat similar documents describing what is needed, so aligning a curriculum to as many as possible of those other resources makes sense. It is also interesting to see that implementation does not necessarily map easily to what curriculum documents describe. It would be interesting to know just how well the content of those sessions actually mapped to curriculum documents, even where session names did not. The discussions may well cover desired territory, but also may be discursive and tangential. Ultimately, assessment practices may dictate what is learned, so mapping further to assessment would add value here.

Reviewer Expertise:

NA

No decision status is available
